# Hybrid the long short-term memory with whale optimization algorithm and variational mode decomposition for monthly evapotranspiration estimation

**DOI:** 10.1038/s41598-022-25208-z

**Published:** 2022-12-01

**Authors:** Tonglin Fu, Xinrong Li

**Affiliations:** 1 School of Mathematics and Statistics, Longdong University, Qingyang, 745000 China; 2 Shapotou Desert Research and Experiment Station, Northwest Institute of Eco-Environment and Resources, Chinese Academy of Sciences, Lanzhou, 730000 China

**Keywords:** Environmental sciences, Hydrology

## Abstract

The sustainability of artificial sand-binding vegetation is determined by the water balance between evapotranspiration (ET) and precipitation in desert regions. Consequently, accurately estimating ET is a critical prerequisite for determing the types and spatial distribution of artificial vegetation in different sandy areas. For this purpose, a novel hybrid estimation model was proposed to estimate monthly ET by coupling the deep learning long short term memory (LSTM) with variational mode decomposition (VMD) and whale optimization algorithm (WOA) (i.e., VMD-WOA-LSTM) to estimate the monthly ET in the southeast margins of Tengger Desert. The superiority of LSTM was selected due to its capability of automatically extracting the nonlinear and nonstationary features from sequential data, WOA was employed to optimize the hyperparameters of LSTM, and VMD was used to extract the intrinsic traits of ET time series. The estimating results of VMD-WOA-LSTM has been compared with actual ET and estimation of other hybrid models in terms of standard performance metrics. The results reveale that VMD-WOA-LSTM provide more accurate and reliable estimating results than that of LSTM, the support vector machine (SVM), and the variants of those models. Therefore, VMD-WOA-LSTM could be recommended as an essential auxiliary method to estimate ET in desert regions.

## Introduction

Evapotranspiration (ET) is a highly nonlinear physical and biological process, which connects the ecological and hydrological processes by the water balance^1,2^. It is the central component of regional water and energy balance, and serves as a significant linkage in the soil–plant-atmosphere (SPA) system^3^. Accurately estimating ET is a critical prerequisite in environmental management^4–6^, especially in desert regions with large areas of artificial sand-binding vegetation, where the sustainability of artificial sand-binding vegetation is determined by the water balance between ET and precipitation^5,7^ . In addition, climate change, especially changes in warming and precipitation patterns, will inevitably have a profound impact on the sustainability of artificial vegetation^7,8^. Different from the natural vegetation, artificial sand-binding vegetation is established with speciall purpose and function, the accurate estimation of ET can provide a reference for understanding the water balance and determining the composition, structure, spatial distribution, and scale of artificial sand-binding vegetation in desert regions^9,10^. However, the application of physically-based methods (e.g., Priestley-Taylor method, Hargreaves method, the corrected FAO-24 Penman method, FAO-56 Penman–Monteith method, etc.) is severely limited due to the lack of required meteorological parameters (e.g. the latent heat of vaporization, solar radiation, relative humidity, air temperature, etc.) in desert regions^4,6,2–12^. Therefore, constructing the other types of data-driven models to obtain accurate estimating results is highly desirable.

Recently, the machine learning (ML) models, including back-propagation neural networks (BPNN)^13^, multi-layer perceptron (MLP)^2^, Multilayer artificial neural networks (MLNN)^6^, support vector machine (SVM)^7,12^ , extreme learning machine (ELM) ^6^, Model tree (MT)^14,15^, random forest (RF)^6^, wavelet neural networks (WNN)^16^, radial basis function (RBF)^17^, etc., have been dramatically employed to estimate evaporation or ET due to its capability of automatically learning features and not requiring any assumptions. As ML models have the defects that the hyperparameters are difficult to adjust by themselves, which significantly decrease the computing accuracy. To overcome the drawbacks of ML models, meta-heuristic algorithms such as flower pollination algorithm (FPA)^6^, firefly algorithm (FFA)^11^, intelligent water drops (IWD) algorithm^12^ , whale optimization algorithm (WOA)^18^, grey wolf optimizer algorithm (GWO)^19,20^ etc., were employed to determine the optimal hyperparameters of ML models. Studies have shown that ML models coupled with meta-heuristic algorithms have higher computing performance than that of single ML models and physically-based methods^12,16,18,21,22^.

As ET is closely affected by the meteorological parameters, soil moisture, and vegetation traits^12^, the measured ET time series taking on many sharp and fluctuating points, which significantly decreased the estimating accuracy^12^. To obtain more credible estimating results, data pre-processing techniques, including Discrete wavelet transform (DWT)^23^, ensemble empirical mode decomposition (EEMD)^14,15^, and variational mode decomposition (VMD)^7,24^ etc., were employed to decompose ET time series frequency into various components and obtain the required information at multiple levels^7,14,23,24^. Literature review shows that data pre-processing techniques hybridized with ML models can significantly improve the model performance^16,25^ . In this regard, Gocić et al.^22^coupled SVM with DWT and firefly algorithm (FFA) to estimate reference ET in Serbia, where FFA was employed to determine the hyperparameters of SVM. The results show that DWT-FFA-SVM is the best estimating method for reference ET estimation. Pammar and Deka^[24]^proposed a hybrid DWT-SVM to estimate the daily pan evaporation in Karnataka, India. The results also confirm that SVM combined with DWT can improve the estimation accuracy. Rezaie-Balf et al.^15^ integrated EEMD with SVM and M5 model tree (M5T) separately to estimate the monthly pan evaporation models of Siirt station and Diyarbakir station in Turkish, and the proposed models presented much higher accuracy. Fu et al.^7^ proposed hybrid models by combining the DWT, EEMD, and VMD with SVM and GWO-SVM separately to estimate the monthly ET. The results indicated that VMD and DWT exhibited better pre-processing performance than that of EEMD, and the estimating accuracy of VMD-GWO-SVM was higher than that of DWT-GWO-SVM and EEMD-GWO-SVM**.**

The previous works are mainly focused on using shallow ML models to estimate ET^2,4,6,7,2–18,21–23^. It is well known that the shallow ML models have drawbacks that cannot sufficiently extract the hidden nonlinear and non-static features from the ET time series^25^. Thus, long short-term memory (LSTM)^3,26^, deep neural network (DNN)^27^, temporal convolution neural network(TCN)^27^, recurrent neural network (RNN)^28^ have been employed to estimate ET or evaporation based on limited meteorological data. E.g., Majhi et al.^3^ used LSTM, MLNN, Hargreaves formula, and Blaney-Criddle formula to estimate daily pan evaporation of Chhattisgarh state in India. The results indicate that LSTM can achieve accurate estimation of evapotranspiration, and has better estimation than other models. Chen et al.^27^estimated the daily reference ET in the Northeast plain of China by using LSTM, DNN, TCN, SVM, RF, Hargreaves model, Ritchie method, Priestley-Talor model, Makkink formula, Romanenko model, and Schendel formula, respectively. The results show that the LSTM, TCN and DNN have better estimation performance than that of the shallow ML models and empirical models in the absence of meteorological parameters. Granata and Di Nunno^28^ used LSTM and NARX to estimate ET of Cypress Swamp and Kobeh Valley in the USA. The results show that deep learning models have higher precision than the shallow ML models due to the high hierarchical structure.

In fact, the hyperparameters of ML models directly determine the computing accuracy, but most data driven models are unable to search the optimal hyperparameters by themselves, and LSTM is no exception. The hyperparameters of LSTM, including the number of hidden layers (HL), number of hidden units (HU), epochs, and learning rate (LR)^26^, significantly affect the estimating performance of LSTM. However, to the best knowledge of the authors, the application of LSTM coupled with meta-heuristic algorithms to estimate evaporation or ET has been very minimal.

## Study area and data

The research was conducted in the southeast margins of Tengger Desert (37°32'N, 105°02'E). The primary landscape type is densely distributed trellis dunes^9,10^. To prevent the harm of sandstorms to the Baotou-Lanzhou railway, the Chinese academy of sciences and relevant units of railway have established artificially re-vegetated belt in 1956a and extended in 1964a, 1981a, and 1987a. Mechanical sand barrier perpendicular to the main wind direction was installed on the mobile dune, straw checkerboards (spacing 1 m × 1 m) were set behind the mechanical sand barrier, and two-year xeristic shrub seedlings were planted in the same configuration in a banded way with plant spacing and row spacing of 1 m × 2 m or 2 m × 3 m under the condition of no irrigation. After more than half a century of succession, the number of natural plant species has increased from 25 to 453, and the vegetation coverage has increased from less than 1% to 42.3%. A biological windbreak sand fixation zone with a length of 16 km and a width of 200-1000 m has been gradually formed. The artificial sand-binding vegetation established in different years (1956a, 1964a, 1981a and 1987a) are distributed on both sides of the railway in parallel, which successfully prevented the damage of wind-blown-sand damage to railway traffic and significantly improved the ecological environment of the study area. As the stability and sustainability of the revegetation depend on the water balance between ET and precipitation^7,9,10^, it is of great theoretical and practical significance to accurately estimate ET for protecting and utilizing artificial sand-binding vegetation^7,10^.

In this study, the monthly ET data measured from January 1991 to December 2018, the data from January 1991 to December 2010 were regarded as the training set, and the rest was used as the testing set. Table 1 shows the main statistical metrics of the monthly ET time series in the study area.

**Table 1:** The statistical characters of the monthly ET time series.

	Data set	Mean	Std.	Skewness	Kurtosis	Minimum	Maximum
ET	Training	221.2187	126.7168	0.246	− 1.233	29.3	488.40
	Testing	182.0594	107.8447	0.381	− 1.064	32.8	424.80
	Total	210.0304	122.7508	0.321	− 1.134	29.30	488.40

## Methodology

### The framework of the proposed models

LSTM is a new time cycle neural network that can overcome the gradient vanishing problem in RNN by adding a chain form of repeating neural network modules to store relevant information^25,26^. It uses the working principle of “two in and two out” to solve the problem of long-order dependency^26^. In this study, LSTM was selected as main modular to estimate the monthly ET duo to the LSTM has the excellent capability of tackling nonlinear patterns among the time series^27,28^. In adition, SVM was also employed to estimate ET since SVM has better adaptability to solve a broader class of nonlinear fitting problems (e.g., estimate ET) than that of other shallow ML models (e.g., BPNN, WNN, ELM, MT, and MLP)^29^ .

As DWT is sensitive to the wavelet basis and the threshold, EMD suffers from an intrinsic drawback of mode mixing^7,24^, and EEMD exists endpoint effects^7^. VMD is a more robust and adaptive data pre-processing method than DWT, EMD, and EEMD^24^, it effectively overcomes the shortcomings of those methods and extract the main variation features from the irregularity and non-stationarity time series^24^. In this study, VMD was employed to extract the main variation features from the ET data set. To compare the denoising performance of VMD, DWT was selected as the reference. The output of DWT, VMD and the collected ET time series were divided into training set and testing set, and fed to ML models to estimate ET, respectively.

**Figure 1:**
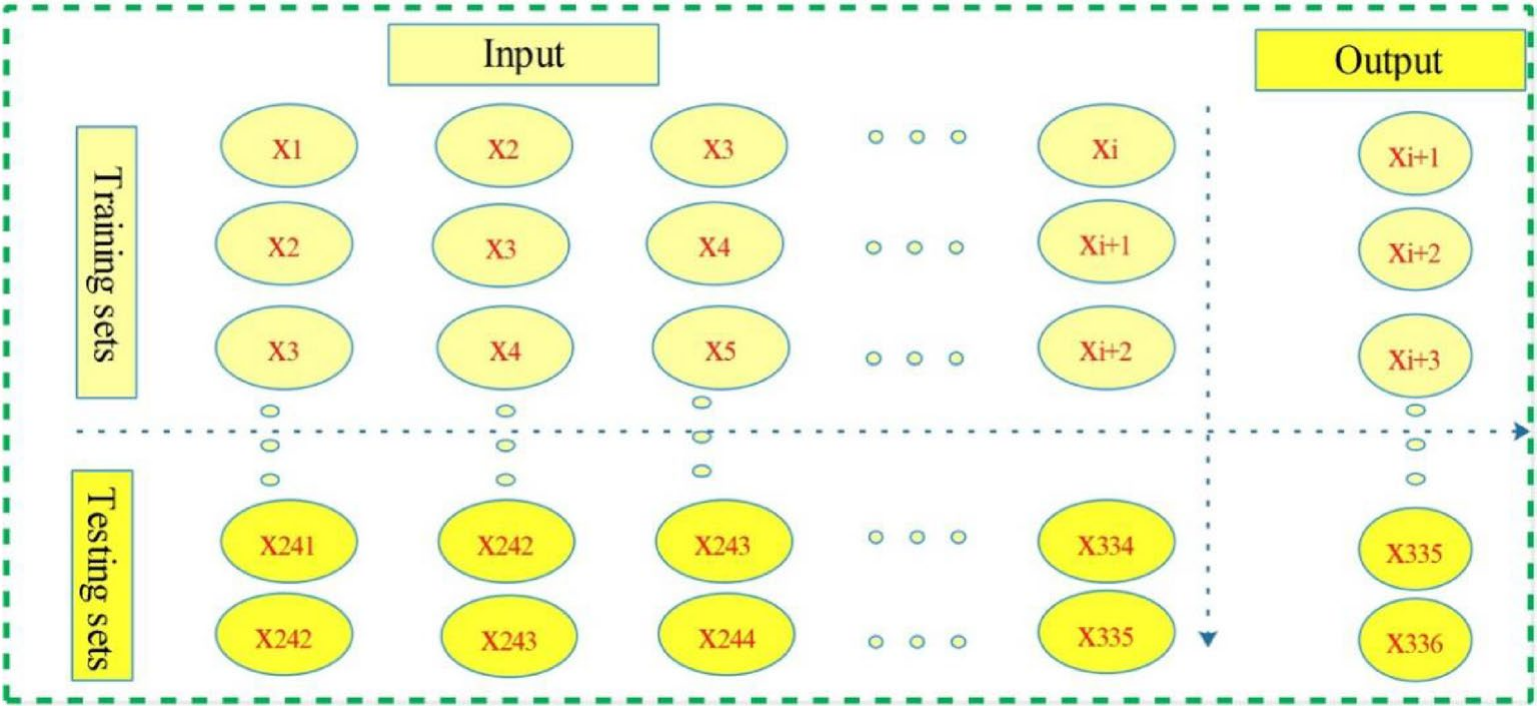
The schematic diagram of LS method.

To improve the training efficiency of ML models, the min–max normalization method^7,22^ was used to normalize the input and output data sets. The input and output sets of the SVM and LSTM were determined by using the longitudinal selection (LS) method^7^, namely, the group of training samples is represented by a vector from $i$ to $k - q$, the training output is denoted as a vector from $i + d - 1$ to $k - q$, the testing output vector is represented by a vector from $k - q + 1$ to $k$, where $i$, $d$, $k$ and $q$ denote the starting point, the input dimension, the size of data set, and the length of the testing output, respectively. Figure 1 shows the schematic diagram of the LS method with $i = 1$, $d = 3$ and $k = 336$.

**Figure 2:**
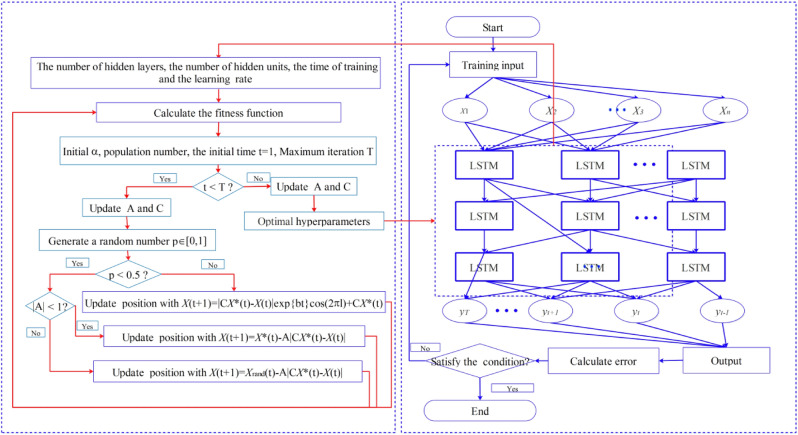
The flowchart of the WOA-LSTM.

WOA is a new efficient and stable meta-heuristic algorithms^18^, which was widely used to solve the nonlinear optimization problems, including optimizing the hyperparameters of shallow ML models for estimation of evaporation or ET. However, no researcher has proposed a hybrid model by coupling LSTM with meta-heuristic algorithms to estimate ET. Thus, the WOA was employed to optimize the hyperparameters of LSTM to obtain more accurately estimating results. Figure 2 shows the flowcharts of WOA-LSTM. The WOA-LSTM coupled with VMD is denoted as VMD-WOA-LSTM.

**Figure 3:**
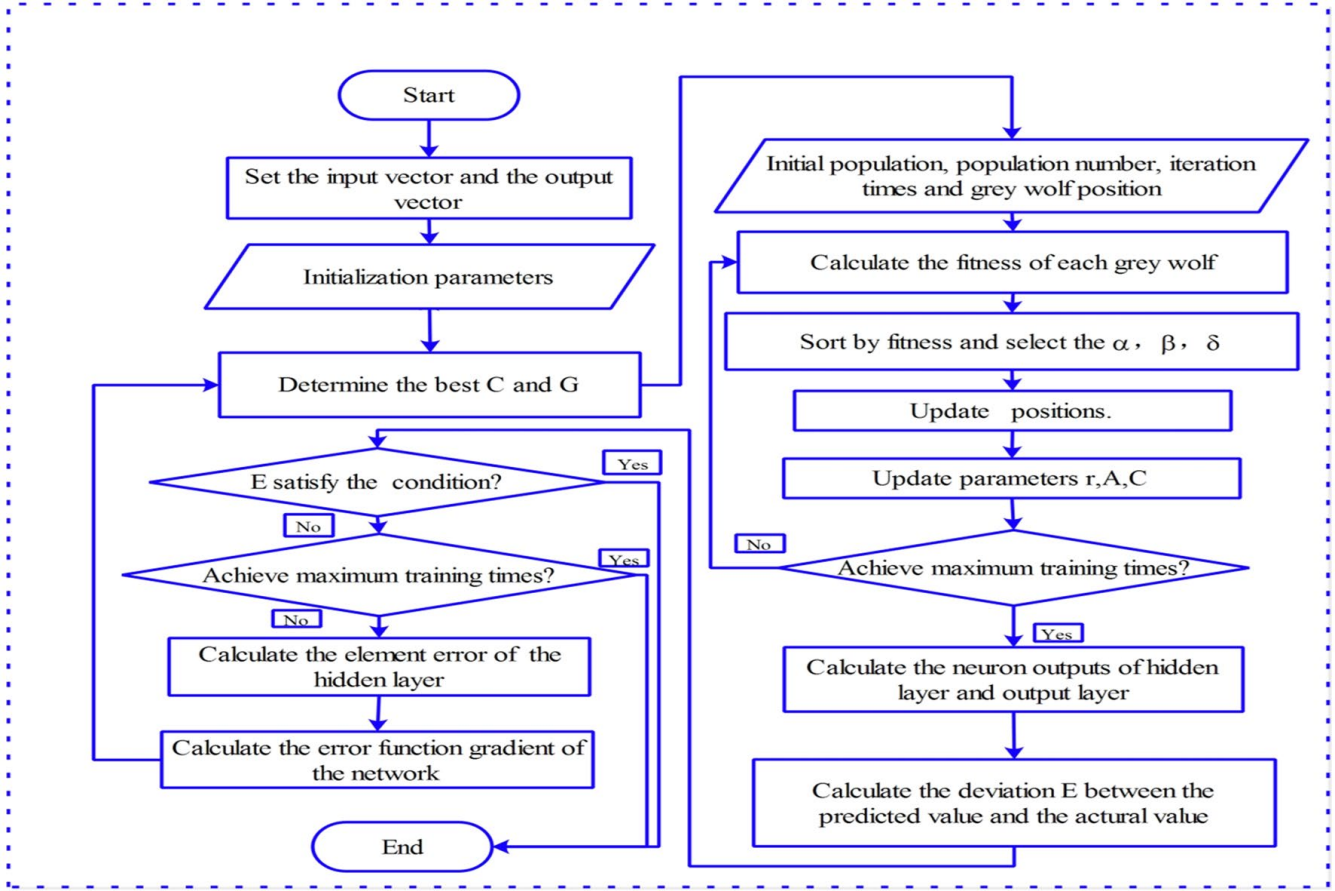
The flowchart of GWO-SVM.

The superiority of VMD-WOA-LSTM was tested by comparing the estimating performance with LSTM, SVM, DWT-LSTM, DWT-SVM, VMD-LSTM, VMD-SVM, WOA-LSTM, GWO-SVM, DWT-WOA-LSTM, DWT-GWO-SVM, and VMD-GWO-SVM, where GWO-SVM indicates that the best C and G of SVM were determined by using GWO algorithm, Fig. 3 show the flowcharts of GWO-SVM. In the numerical simulation processes, the mean absolute percentage error (MAPE) was employed as the fitness function to determine the optimal hyperparameters of ML models. It should be noted that the mathematical principles of the methods used in this paper, including DWT, VMD, SVM, LSTM, GWO and WOA, can be found in relevant literatures, so the mathematical description of these methods is omitted.

### Metrics for performance evaluation

The error analysis is performed by using the evaluation metrics, including the mean absolute error (MAE), MAPE, normalized mean squared error (NMSE), root mean squared error (RMSE), and Nash–Sutcliffe coefficient of efficiency (NSCE). The definitions of those evaluation metrics are as follows:

1 $$ MAE = \frac{{1}}{n}\sum\limits_{i = 1}^{n} {\left| {y_{i} - \widehat{y}_{i} } \right|} $$

2 $$ MAPE = \frac{{1}}{n}\sum\limits_{i = 1}^{n} {\left| {\frac{{y_{i} - \widehat{y}_{i} }}{{y_{i} }}} \right|} \times 100\% $$

3 $$ NMSE = \frac{{1}}{n}\sum\limits_{i = 1}^{n} {\left( {\frac{{y_{i} - \widehat{y}_{i} }}{{y_{i} }}} \right)^{2} } $$

4 $$ RMSE = \sqrt {\frac{{1}}{n}\sum\limits_{i = 1}^{n} {\left( {y_{i} - \widehat{y}_{i} } \right)^{2} } } $$

5 $$ NSCE = 1 - {{\sum\limits_{i = 1}^{n} {\left( {y_{i} - \widehat{y}_{i} } \right)^{2} } } \mathord{\left/ {\vphantom {{\sum\limits_{i = 1}^{n} {\left( {y_{i} - \widehat{y}_{i} } \right)^{2} } } {\sum\limits_{i = 1}^{n} {\left( {y_{i} - \overline{y} } \right)^{2} } }}} \right. \kern-\nulldelimiterspace} {\sum\limits_{i = 1}^{n} {\left( {y_{i} - \overline{y} } \right)^{2} } }} $$

where $y_{i}$ and $\widehat{y}_{i}$ denote the desired and estimated result.

The MAE, MAPE, NMSE, and RMSE are generally used to measure the deviation between the desired and estimated result, the estimating performance is better when the values of these evaluation metrics are smaller. As MAPE is relatively more stable among these criterions, and NSCE is generally used to determine model efficiency in the hydrological field (NSCE close to 1 indicate the proposed model has good fitness). Therefore, MAPE and NSCE were selected as the main benchmarks to evaluating the model performance of the proposed models.

### Parameter settings

The estimating performance of different models is primarily determined by the parameters selection. Table 2 shows the key parameters of the DWT and VMD. The number of search agents and the maximum iterations of WOA and GWO algorithms were 5 and 100, respectively. The mini-batch size of all the LSTM-based models was 128. The range of HL, HU, Epochs and LR of WOA-LSTM were [1, 200], [1, 200], [10, 100], and [0.001, 0.01], respectively. As for GWO-SVM, the range of C and G were [0.01, 100].

**Table 2:** The experiment parameters and denoising performance of DWT and VMD.

DWT	db4 with	VMD	
Threshold method	Rigorous sure	The balance parameter parameterata fidelity constraint	$\alpha = 0.05$
Threshold rule	Soft	The step of the dual ascent	$\tau = 0$
Noise structure	Unscaled wite noise	The number of modes	K = 5
SNR	40.8201	SNR	42.6451
RMSE	2.2127	RMSE	1.7934

## Results

**Figure 4:**
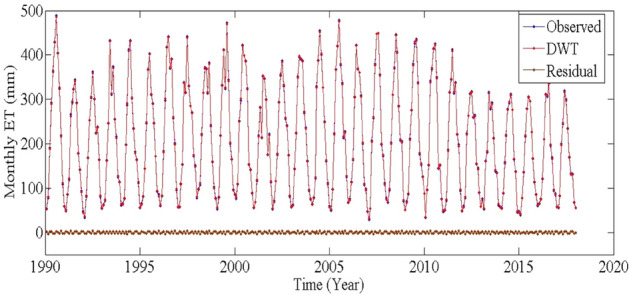
The results of DWT.

**Figure 5:**
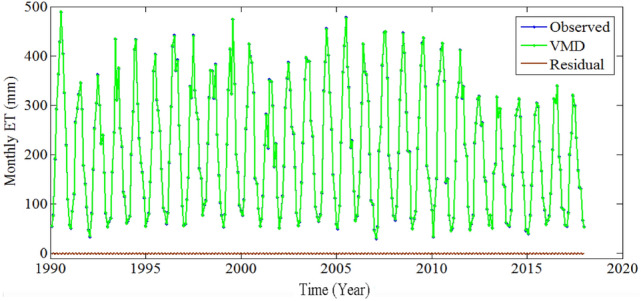
The results of VMD.

Figures 4 and 5 show the results of DWT and VMD. As shown in Figs. 4 and 5, the collected monthly ET time series have many sharp and fluctuating points, which will affect the estimating performance. To resolve these problematic patterns in ET estimating processes, the db4 with level 1 and VMD with K = 5 were finalized and employed to denoise the steady ET time series. The residual time series shows the low amplitude and high-frequency fluctuation, suggesting that the white noise was eliminated from the original ET data set. Table 2 shows that the denoising performance of VMD (with SNR = 42.6451 and RMSE = 1.7934) is more efficient than that of DWT (with SNR = 40.8201 and RMSE = 2.2127). In fact, DWT has the the disadvantages that the wavelet basis should be selected in advance, and it is also sensitive to the selection of threshold. Therefore, VMD has better denoising performance than that of DWT in this study.

As mentioned, LSTM and SVM were used to estimate the monthly ET, respectively. For each fixed input dimension d in d = 2, 3, …, 16, the minimum MAPE was selected from 5 replications, and the optimal hyperparameters of the WOA-LSTM and GWO-SVM were recorded according to the minimum MAPE. Table 3 shows the optimal input features and hyperparameters of proposed models. As shown in Table 3, the MAPE of each LSTM-based model is less than that of SVM-based, suggesting that the deep learning models significantly outperformed the shallow ML models; The MAPE of single ML model was decreased by coupling ML model with the data pre-processing techniques, suggesting that the performance of ML model can be improved by using the data pre-processing; The MAPE of the hybrid models with optimal parameters are all smaller than that of the ML models with default parameters, indicating that ML models integrated with meta-heuristic algorithms have moderately higher computing performance than that of the ML models with default parameters; The MAPE of the hybrid DWT-GWO-SVM, DWT-WOA-LSTM, VMD-GWO-SVM, and VMD-WOA-LSTM models are smaller than that of LSTM, SVM, DWT-LSTM, DWT-SVM, VMD-LSTM, VMD-SVM, WOA-LSTM, and GWO-SVM, suggesting that the performance of ML model can be significantly improved by using the data pre-processing and meta-heuristic algorithm.

**Table 3:** The experiment parameters of the proposed model.

Parameters	LSTM	DWT-LSTM	VMD-LSTM	WOA-LSTM	DWT-WOA-LSTM	VMD-WOA-LSTM
Input features	10	12	12	12	12	12
No. of HL	200	200	200	90	159	70
No. of HU	200	200	200	140	144	79
Epochs	100	100	100	47	51	64
LR	0.005	0.005	0.005	0.0098	0.0085	0.0068
Batch siz	128	128	128	128	128	128
MAPE	28.18%	25.55%	27.17%	18.97%	19.72%	18.90%

Parameters	SVM	DWT-SVM	VMD-SVM	GWO-SVM	DWT-GWO-SVM	VMD-GWO-SVM
Input features	3	3	3	4	4	3
C	1	1	1	55.4135	68.9681	86.0928
G	0.3333	0.3333	0.3333	0.7248	1.2431	0.7648
MAPE	28.42%	27.94%	27.46%	24.14%	23.69%	23.21%

**Figure 6:**
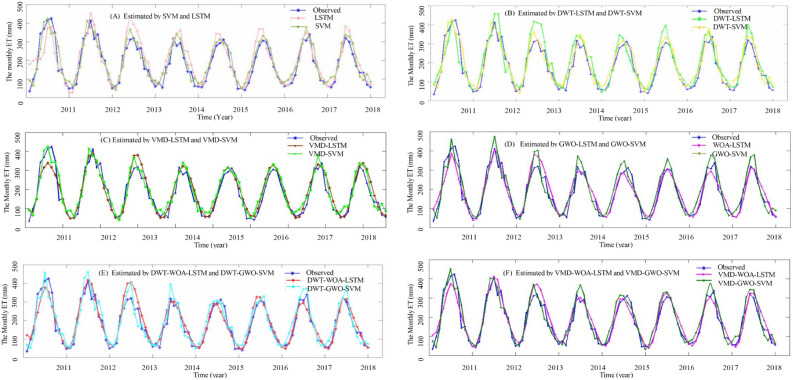
ET estimation using (**A**) LSTM and SVM, (**B**) DWT-LSTM and DWT-SVM, (**C**) VMD-LSTM and VMD-SVM, (**D**) WOA-LSTM and GWO-SVM, (**E**) DWT-WOA-LSTM and DWT-GWO-SVM, (**F**) VMD-WOA-LSTM and VMD-GWO-SVM.

The estimating results of the ML models with the minimum MAPE and the optimal hyperparameters are presented in Fig. 6. Figure 6 shows that the results of all the proposed models are consistent with most of the points in the middle range, but the starting point and extreme values are overestimated; The monthly ET time series have a period of 12 months, the optimal input dimensions of LSTM, DWT-LSTM, VMD-LSTM, WOA-LSTM, DWT-WOA-LSTM,and VMD-WOA-LSTM are approximately equal to the period of the ET time series (Table 3), suggesting that LSTM can take full advantage of historical data patterns and effectively overcome the drawbacks of shallow ML models. Overall, the hybrid DWT-GWO-SVM, DWT-WOA-LSTM, VMD-GWO-SVM, and VMD-WOA-LSTM models have better estimating performance than that of other proposed models.

Table 4 shows the evaluation metrics of each model in the training and testing stages, where the evaluation metrics average of the ML models optimized by meta-heuristic algorithms are marked in bold**.** As shown in Table 4, the model performance of DWT-LSTM, DWT-SVM, VMD-LSTM, and VMD-SVM models is better than that of LSTM and SVM in testing stage (Table 4), suggesting that the data pre-processing is an aid to enhance the estimating performance of ML models. As shown in Table 4, the evaluation metrics including MAE, MAPE, NMSE, and RMSE of WOA-LSTM and GWO-SVM are all smaller than that of LSTM, SVM, DWT-LSTM, DWT-SVM, VMD-LSTM and VMD-SVM, and NSCE increased to varying degrees, indicating that the estimating performance of LSTM and SVM are significantly improved when the hyperparameters of LSTM and SVM are optimized by using meta-heuristic algorithms.

**Table 4:** The evaluation metrics in the training and testing stages.

Model	Training					Testing				
	MAE	MAPE (%)	NMSE	RMSE	NSCE	MAE	MAPE (%)	NMSE	RMSE	NSCE
LSTM	14.4930	8.97	0.0140	33.0085	0.9767	32.9698	28.18	0.3047	41.0264	0.8738
SVM	33.9835	18.89	0.0909	45.5818	0.8700	33.4904	28.42	0.1740	40.3111	0.8588
DWT-LSTM	12.1676	7.01	0.0167	31.3245	0.9545	32.5677	25.55	0.1187	36.4157	0.8682
DWT-SVM	32.8495	18.23	0.0850	44.2475	0.8775	32.9807	27.94	0.1665	39.6668	0.8633
VMD-LSTM	12.3580	6.99	0.0120	31.4618	0.9817	35.7024	27.17	0.1261	39.8266	0.8843
VMD-SVM	34.0165	19.10	0.0937	45.4876	0.8705	33.4178	27.46	0.1760	40.2667	0.8591
WOA-LSTM	**12.8634**	**5.74**	**0.0162**	**29.7665**	**0.9845**	**26.7628**	**18.98**	**0.1020**	**40.6856**	**0.8851**
GWO-SVM	**25.3157**	**15.47**	**0.0474**	**34.5420**	**0.9254**	**32.7588**	**24.56**	**0.1303**	**40.4901**	**0.8575**
DWT-WOA-LSTM	**10.3077**	**5.43**	**0.0104**	**28.5546**	**0.9966**	**27.0411**	**18.90**	**0.1257**	**35.3072**	**0.8578**
DWT-GWO-SVM	**22.3948**	**13.76**	**0.0346**	**28.8268**	**0.9479**	**32.6383**	**23.77**	**0.1021**	**40.2469**	**0.8593**
VMD-WOA-LSTM	**11.3376**	**5.46**	**0.0102**	**28.7892**	**0.9805**	**26.8135**	**18.72**	**0.1048**	**37.4979**	**0.8917**
VMD-GWO-SVM	**29.7499**	**16.98**	**0.0544**	**41.4806**	**0.8923**	**25.2081**	**23.22**	**0.1122**	**37.8756**	**0.8754**

The evaluation metrics of SVM integrated with GWO are all smaller than that of the LSTM coupled with data pre-processing techniques; The MAPE of hybrid VMD-GWO-SVM is smaller than that of other SVM-based models and LSTM, DWT-LSTM, VMD-LSTM models (Table 4), suggesting that shallow ML models integrated with data pre-processing techniques and meta-heuristic algorithms can be employed to estimate ET. Whereas, the model performance of shallow ML models still needs to be improved. As shown in Table 4, the deep learning models significantly outperformed the shallow ML models, and the model performance of hybrid WOA-LSTM, DWT-WOA-LSTM, and VMD-WOA-LSTM are better than that of the SVM-based hybrid models. Compared with VMD-GWO-SVM, the the MAPE of DWT-WOA-LSTM was decreased from 23.22% to 18.90%, and NSCE was improved from 0.8754 to 0.8578; The MAPE of VMD-WOA-LSTM was decreased from 23.22% to 18.72%, and NSCE was improved from 0.8754 to 0.8917. Those results indicating that the hybrid DWT-WOA-LSTM and VMD-WOA-LSTM models have excellent capability in estimating the monthly ET over other proposed methods. The MAPE of the hybrid VMD-WOA-LSTM models is the smallest among the proposed models, indicating that VMD-WOA-LSTM is the most precise model to estimate the monthly ET among the proposed models. Therefore, VMD-WOA-LSTM could be recommended as an essential auxiliary method to estimate ET in desert regions.

## Discussion

As mentioned, the hybrid models based on ML models, data pre-processing techniques and meta-heuristic algorithms were proposed to estimate ET in the study area. Generally, constructing the hybrid ML models is a complex and time-consuming procedure^6,14–18^. However, the hybrid ML models have higher accuracy than the physically-based methods^20–23^. Therefore, using the hybrid ML models to precisely estimate the ET is a core issue in hydrology and ecology.

The deep learning models generally outperformed the shallow ML models^3^. However, only using ML models to estimate ET may lead to large errors when the data pre-processing is not performed^7,14–16^. As show in Figs. 4 and 5, the collected ET time series exhibit the irregularity and non-stationarity since ET is determined by the meteorological factors and closely related to the soil moisture, geomorphological and vegetation traits in desert regions. Thus, data preprocessing technique should be used to to extract the main variation features from the collected ET time series for obtaining more credible estimating results. The data preprocessing methods such as DWT and VMD can be applied to eliminate the noise from the non-linear and non-stationary time series. Whereas, DWT is sensitive to the selection of threshold, VMD is a more robust and effective data preprocessing method than the empirical mode decomposition (EMD), and EEMD, which can be used to remove the noise from time series by decomposing the nonlinear and nonstationary signal into several band-limited intrinsic mode functions (IMFs)^7,24^. Therefore, the denoising performance of DWT is worse than that of VMD (Table 2).

Literature review shows that the ML models integrated with data pre-processing technique is an effective strategy for obtaining more credible estimating results^7,21–23^, the results in our research also confirm thi s conclusion. As shown in Tables 3 and 4, the the evaluation metrics average of the ML models integrated with DWT or VMD are all smaller than that of of single SVM and LSTM models. Therefore, extracting useful features by using data preprocessing technique is necessary for more credible estimating results (Supplementary File 1).

The estimating performance of the ML models can also be significantly improved by coupling ML models with meta-heuristic algorithms. To a certain extent, it is more important to obtain the optimal hyperparameters of a ML model than to select the appropriate data preprocessing technique. As shown in Tables 3 and 4, the estimating performance of ML models hybridized with data preprocessing technique were significantly improved when the hyperparameters of LSTM and SVM were optimized. Compared with DWT-SVM, VMD-SVM, DWT-LSTM, and VMD-LSTM, the positive evaluation metrics of DWT-GW0-SVM, VMD-GWO-SVM, DWT-WOA-LSTM and VMD-WOA-LSTM decreased, and the negative indicators increased to varying degrees. Thus, the selection of hyperparameters for ML models was crucially important to improve the estimating performance^6,7,21–29^ .

Furthermore, as concluded, the main objectives of this study was to propose a novel hybrid model to estimate ET by integrating the LSTM with WOA and data preprocessing techniques, including DWT and VMD. The estimating performance of VMD-WOA-LSTM was compared with other hybrid models in terms of standard performance metrics. The performance comparison of the proposed models demonstrated that the hybrid VMD-WOA-LSTM model performed better than other ML-based models to estimate the monthly ET in the southeast margins of Tengger Desert.

## Conclusions

In this study, the hybrid models based on the VMD, WOA, and LSTM were proposed to estimate ET using the time series analysis methods, where VMD was used to extract the intrinsic traits of ET time series, and WOA was employed to optimize the hyperparameters of LSTM. The model performance was compared with actual ET and estimation of other hybrid models in terms of MAE, MAPE, NMSE, RMSE, and NSCE. The results indicate that VMD-WOA-LSTM has more accurate estimating results than other ML-based models, which can be recommended as an essential auxiliary method to estimate ET in desert regions.

## Supplementary Information

Supplementary Information.

## Data Availability

All data analyzed or generated during this study are included in the Supplementary Information, and are available from the corresponding authors upon reasonable request.
